# Towards universal health coverage: Data for determinants of immunization coverage of *Pneumococcal* and Rota virus vaccines among under five children in Busolwe Town Council, Butaleja District, Eastern Uganda

**DOI:** 10.1016/j.dib.2019.104269

**Published:** 2019-07-15

**Authors:** Brenda Wafana Nabwana, Sylvia Sidney Namayanja, Collette Kemigisha, Erina Kisakye, Amos Kuddiza Kusetula, Silvester Wakabi, Ivan Wambi, Innocent Musiime, Rebecca Nekaka, Yahaya Gavamukulya

**Affiliations:** a Department of Community and Public Health, Faculty of Health Sciences, Busitema University, P.O. Box, 1460 Mbale, Uganda; b Busolwe General Hospital, Butaleja District Local Government, Butaleja District, Uganda; c Department of Biochemistry and Molecular Biology, Faculty of Health Sciences, Busitema University, P.O. Box, 1460 Mbale, Uganda

**Keywords:** Immunization coverage, PCV, Rotavirus vaccine, Under five children (U5C), Butaleja, Eastern Uganda

## Abstract

The data described stipulates the factors influencing the immunization coverage of *Pneumococcal and Rota Virus Vaccines* among under five children (U5C) in Butaleja district in Eastern Uganda. The data was obtained in three major sections of demographic characteristics, knowledge, and attitude and perceptions of care takers of U5C on immunization. Both qualitative and quantitative types of data obtained from Primary and Secondary data sources are presented. The Primary sources included administration of questionnaires to the caretakers of U5C in communities surrounding different health centers in Butaleja district. The secondary source of data was majorly the Health Management Information Systems (HMIS) records of Busolwe District Hospital. The data includes raw data from individual participants in form of Google forms portable document format, the consolidated raw data from all the participants in Microsoft excel format, as well as raw data from secondary HMIS record on immunization coverage in form of Microsoft excel format. The data provides a general outlook on the state of Butaleja district in terms immunization coverage of Pneumococcal and Rota Virus Vaccines. The data can be useful in taking action to decrease the burden of vaccine preventable diseases in Butaleja and elsewhere in similar settings. The data described is freely available in the Mendeley Data repository at the following site: https://doi.org/10.17632/zr2w886dg2.1 (Nabwana et al., 2019).

**Table:** Specifications table

Subject area	*Health Sciences, Medical Sciences*
More specific subject area	*Immunization, Public Health*
Type of data	*Microsoft Excel csv., Microsoft Excel Macros enabled files, PDF files*
How data was acquired	*Online Researcher Administered Survey accessible at (*https://forms.gle/PCi5rbK1mt5tgzhA8*)*
Data format	*Raw and filtered*
Experimental factors	A cross sectional study design was employed. Demographic and socio-economic characteristics were some of the key concept variables and purposive sampling was preferred. Ethical approval and Permission to conduct the study were obtained from the relevant University and District authorities. Care takers of U5C in Butaleja District who gave an informed consent were included in the study.
Experimental features	Informed consent was sought from the participants prior to participation in the data collection process. An online survey was administered through Google forms to recruited participants.
Data source location	Busolwe Town Council, Butaleja District - Eastern Uganda; Busitema University Faculty of Health Sciences, Mbale – Eastern Uganda
Data accessibility	All the reported datasets can be accessed via the Mendeley Data Repository at (https://doi.org/10.17632/zr2w886dg2.1) [1]

**Table:** 

**Value of the data** • The data is useful to governments in assessment of the immunization coverage and utilization of the vaccines by citizens. • The community members benefit from this data in that feasible solutions can be accorded with respect to evidence-based information on declining trend of immunization. • The data can be used as a reference for comparative studies in similar settings. • The data can help to guide resource allocation and direction of work plan especially pertaining immunization of children. • The data can be useful in evaluation of health indicators such as utilization of immunization services by the line ministries. • The data collection tools can be used in conducting studies in other locations or on other diseases • This data can be used to determine possible trend of immunization coverage for other vaccine preventable diseases like Hepatitis B or vaccinations that require multiple dosages.

## 1 Data

The data described includes raw data from individual participants in form of Google forms portable document format (PDF), the consolidated raw data from all the participants in Microsoft excel format, as well as raw data from secondary HMIS record on immunization coverage in form of Microsoft excel format.

a. Individual responses for the determinants of Immunization Coverage in Busolwe 2019 - Google Forms.pdf [1].
b. Consolidated data for determinants of Immunization Coverage in Busolwe 2019.csv [1].
c. Raw datasets for the secondary HMIS data on immunization.xlsm [1].

## 2 Experimental design, materials, and methods

### 2.1 Area of study

The study from which the data was obtained was carried out in Butaleja District in Eastern Uganda which is bordered by Budaka and Kibuku districts in the North, Mbale in the East, Tororo district in the South East and Namutumba in the West. Butaleja district has a total population of 244153 people of which 119466 (48.9%) are males and 124687 (51.1%) females according to the national population census 2014 [2]. The Busolwe General Hospital has a catchment population of 42298 people, with women in childbearing age being 8544, with number of pregnancies being 2114, number of live births 2051; number under five years is 8544.

### 2.2 Target population

The study from which the data was obtained targeted the caregivers (primary care givers or parents) of U5C in homes in villages in the hospital's catchment area. Parent(s) and/or caretaker to the U5C who refused to give informed consent were excluded.

### 2.3 Study design

The study from which the data was obtained followed a cross sectional study design to study representative samples of a population. Mixed qualitative-quantitative methods were employed using the questionnaires to the caretakers and more information was obtained from the HMIS records.

### 2.4 Sample size determination

The minimum sample size was determined using the Cochran's formula N = (1.96)^2^pq/d^2^, with a confidence level of approximately 95% (1.96).

Where, N = required sample size, P = proportion of population having the characteristics considering recent studies, q = (1-p) and d= ( ±5%) degree of precision. Therefore, considering findings from a current study on Knowledge and Perception of Caregivers about Risk Factors and Manifestations of Pneumonia among Under Five Children in Butaleja District, Eastern Uganda [3],p = 53.7, q = 1–0.537, d = 5/100 = 0.05.Thus, N = [(1.96)2 × 0.537 × 0.463]/(0.05)^2^ = 0.9551/0.0025 = 382 participants. In order to reduce errors, the sample population was enlarged from 382 participants to 434 participants.

### 2.5 Data collection

An interviewer administered questionnaire was used to assess the perceptions and attitudes of the different correspondents towards the immunizable diseases as well as the factors associated with the immunization coverage in Butaleja district. A Google form (Determinants of Immunization Coverage in Busolwe 2019 - Google Forms Questionnaire.pdf [1]) was created and used to administer the questionnaire with datasets directly filled to Excel worksheets. The questionnaire was pretested and validated among 2nd year Medical and Nursing students at BUFHS. Secondary data was obtained from the Busolwe district HMIS records to determine the number of people who immunized fully.

### 2.6 Data storage

The raw data collected on questionnaires (Google forms) was automatically uploaded and securely stored online, and access to it was limited to only 3 administrators.

### 2.7 Data analysis

The data can be analyzed by use of the compiled datasets to assess the concept variables, correlations, tendencies, among others. Excel and STATA programs can be majorly used in the data analysis. The analyzed data/information can be presented in form of statistical tables, charts, and generalized figures, with interpretive descriptions of the information.

## Consent

Written informed consent from caretakers of the U5C was obtained before they participated in the study. Participants were informed that their privacy and confidentiality would be respected and that there was no potential harm associated with participating in the study. It was made clear to the participants that participation in the study was voluntary and that they were free to opt out of the study at any time without any negative consequences.

## Ethical approval

The study and all the protocols from which the data was obtained were approved and cleared by the Busitema University Faculty of Health Sciences Higher Degrees and Research Committee (BUFHS-HDRC) as part of the Community Based Education, Research and Services (COBERS) Program for the 2018/2019 Academic year under the Course of Community Diagnosis and Communication Projects. Permission to conduct the study was sought from the District Health Officer Butaleja and the Medical Superintendent of Busolwe Hospital. The Chief Administrative Officer (CAO), community leaders and the members of the community consented to the research activities for the data collection; and in this all the participants signed a consent form which clearly stated their rights and the boundaries of the research. All the personal data was kept confidential and participant items under lock and key.
